# The Innocuousness of Nitrous-Oxide Gas, and the Possibility of Producing Prolonged Anæsthesia by Means of This Gas

**Published:** 1880-12

**Authors:** P. W. Van Peyma


### THE INNOCUOUSNESS OF NITROUS-OXIDE GAS, AND THE POSSIBILITY OF PRODUCING PROLONGED ANÆSTHESIA BY MEANS OF THIS GAS.

FROM THE DUTCH BY P. W. VAN PEYMA, M. D.

THESE facts have been experimentally proved by P. Best. As is well known nitrous-oxide gas, the anæsthetic properties of which were discovered by Davy in the close of the preceding century, has in later times been employed by dentists in the causation of a complete, but very transient anæsthesia. For this purpose it is necessary to employ unmixed nitrous-oxide gas, and on account of the great danger of suffocation the inhalation can only be continued for a few seconds.

Paul Best, who is well known in consequence of his beautiful experiments, on the influence upon the human body, of increased and diminished atmospheric pressure, employs his apparatus for rarified air in his experiments with nitrous-oxide gas. In company with a dog that was to be rendered insensible, he placed himself in a chamber where the atmospheric pressure was increased one-fifth. In this atmosphere he now caused the dog to inhale a mixture of nitrous-oxide gas, five-sixths, and oxygen one-sixth. With the usual atmospheric surroundings this mixture would not produce anæsthesia. That it must, then, be employed unmixed, indicates that only under the pressure of one atmosphere into the blood can it pass in an adequate quantity. As, however, the atmospheric pressure throughout the chamber is six-fifths, the inhalation of the mixture has the same effect as that of unmixed nitrous-oxide gas in an atmospheric pressure of one; for now a sufficient quantity of oxygen continues to pass into the blood.

After one or two minutes of restlessness the dog becomes insensible; the cornea can be touched without causing a movement of the lid; pinching and cutting gave rise to no reflex movements or indications of pain. On the other hand the respiratory and cardiac movements continue strong and regular. This condition can be continued for an hour without anything remarkable being observed. The blood remains red in color, the temperature normal. Peripheral nerve excitation which, as has been claimed, is no longer reflected to the animal functions and the sensibility continues to exercise its stimulating effect upon cardiac and respiratory movements.

If we remove the inhaler in which is contained the nitrous-oxide gas, the dog after three or four inhalations of pure air regains sensation, volition and motion, and no indications of ill-effects are apparent. Especially in this regard does the action of nitrous-oxide gas differ from that of chloroform. Chloroform does certainly produce more permanent chemical changes in the blood and tissue; nitrous-oxide gas is eliminated from the blood very rapidly after having but transiently paralyzed the sensorium and the reflex centre of voluntary motion.

The facts and opinions brought forward by Best are extremely clear and simple. He, however, calls attention to the fact that his experiments upon dogs remain to be supplemented by similar observations upon man, although the countless observations of dentists leave little room for doubt as to the harmlessness of nitrous-oxide gas. A second consideration is the difficulty of being obliged to operate, or at least in general, to anæsthetize in a chamber of compressed air. The application of these noteworthy experiments of Best will thus remain limited, but may be important particularly in the department of operative surgery.—*Weekblad van het Nederlandschr. Tydschrift von Geneeskunde, 1879.*

